# Microbiota-derived butyrate is an endogenous HIF prolyl hydroxylase inhibitor

**DOI:** 10.1080/19490976.2021.1938380

**Published:** 2021-06-30

**Authors:** Ruth X. Wang, Morkos A. Henen, J. Scott Lee, Beat Vögeli, Sean P. Colgan

**Affiliations:** a Department of Medicine, Mucosal Inflammation Program, University of Colorado Anschutz Medical Campus, Aurora, CO, USA; b School of Medicine, Medical Scientist Training Program, University of Colorado, Aurora, CO, USA; c Department of Biochemistry and Molecular Genetics, University of Colorado Anschutz Medical Campus, Aurora, CO, USA; d Department of Pharmaceutical Organic Chemistry, Mansoura University, Mansoura, Egypt

**Keywords:** Microbiota, short-chain fatty acids, butyrate, hypoxia-inducible factor, prolyl-hydroxylase, inhibition

## Abstract

The gut microbiota is essential for human health. Microbial supply of short-chain fatty acids (SCFAs), particularly butyrate, is a well-established contributor to gut homeostasis and disease resistance. Reaching millimolar luminal concentrations, butyrate is sequestered and utilized in the colon as the favored energy source for intestinal epithelia. Given the steep oxygen gradient across the anoxic lumen and the highly oxygenated lamina propria, the colon provides a particularly interesting environment to study oxygen sensing. Previous studies have shown that the transcription factor hypoxia-inducible factor (HIF) is stabilized in healthy colonic epithelia. Here we show that butyrate directly inhibits HIF prolyl hydroxylases (PHDs) to stabilize HIF. We find that butyrate stabilizes HIF *in vitro* despite eliminating β-oxidation and resultant oxygen consumption. Using recombinant PHD protein in combination with nuclear magnetic resonance and enzymatic biochemical assays, we identify butyrate to bind and function as a unique, noncompetitive inhibitor of PHDs relative to other SCFAs. Butyrate inhibited PHD with a noncompetitive K_i_ of 5.3 ± 0.5 mM, a physiologically relevant concentration. We also confirm that microbiota-derived butyrate is necessary to stabilize HIF in mice colonic tissue through antibiotic-induced butyrate depletion and reconstitution experiments. Our results suggest that the co-evolution of mammals and mutualistic microbiota has selected for butyrate to impact a critical gene regulation pathway that can be extended beyond the mammalian gut. As PHDs are a major target for drug development in the stabilization of HIF, butyrate holds great potential as a well-tolerated endogenous inhibitor with far-reaching therapeutic impact.

## Introduction

Short-chain fatty acids (SCFAs) produced by the intestinal microbiota through anaerobic fermentation of undigested fiber have multiple roles within the human gut.^1^ Energy procurement depends on the metabolism of SCFAs, which also includes acetate, propionate, butyrate, and low amounts of valerate and hexanoate, through β-oxidation and contributes up to 15% of the host total daily caloric requirement.^2^ Total SCFAs concentrations can reach up to 150 mM in the colon.^1,3^ Butyrate is also a potent histone deacetylase (HDAC) inhibitor that regulates a plethora of intestinal genes.^4–6^ Together, butyrate fundamentally shapes the gut mucosa as both a transcriptional regulator and as an essential substrate for energy metabolism. Decreases in butyrate-producing bacteria and butyrate are key hallmarks of the dysbiosis seen in intestinal diseases.^7^

The relationship between intestinal butyrate and hypoxia-inducible factor (HIF) lies at the intersection of metabolism and gene regulation. Due to the steep oxygen gradient that exists across the anoxic lumen and the highly oxygenated lamina propria, the intestinal mucosa exists in a state of particularly low pO_2_ at baseline, a phenomenon termed “physiologic hypoxia.”^8^ Under such conditions, intestinal epithelial cells (IECs) that line the colon manifest stabilization of hypoxia-inducible factor (HIF). HIF is a master transcriptional regulator of numerous genes important to processes that include erythropoiesis, angiogenesis, energy metabolism, and inflammation.^9^ In normoxia, HIF-α subunits are degraded in an oxygen-dependent manner. When oxygen is limited, HIF-α is stabilized and forms a heterodimeric complex with HIF-1β in the nucleus to bind hypoxia responsive elements in the promoter region of hundreds of target genes.^10,11^ Three HIF-α isoforms (HIF-1α, HIF-2α, and HIF-3α) exist, but HIF-1α and HIF-2α are the best studied, and exhibit similar structures and function with unique and redundant targets.^9^ HIF-α stability is intimately controlled by oxygen levels, increasing slowly between atmospheric to 6% and then exponentially rising as oxygen levels approach 0.5%.^10^ The oxygen-sensitive nature of HIF proteins are reliant on HIF prolyl hydroxylases (HPHs), also known as prolyl hydroxylase domain (PHD) enzymes, which are primed to sense oxygen availability to provide exquisitely specific control of HIF stabilization, as any decrease in oxygen below atmospheric increases PHD enzymatic activity.^11^ PHDs belong to the superfamily of iron and 2-oxoglutarate (2-OG) dependent dioxygenases that utilize molecular oxygen to hydroxylate proline residues within the oxygen-dependent degradation domain (ODD) of HIF-α for recruitment of the von Hippel-Lindau tumor suppressor (pVHL), the recognition element of the E3 ubiquitin ligase that polyubiquitinates HIF-α for proteasomal degradation.^9^ Importantly, β-oxidation of butyrate for energy provision, through forming acetyl-CoA that enters the tricarboxylic acid (TCA) cycle to produce reducing equivalents that drive the electron transport chain to ultimately generate ATP, accounts for greater than 70% of cellular oxygen consumption in the distal colon, and this depletion of oxygen by butyrate is demonstrated to stabilize HIF.^12,13^

Given the strong association between the low pO_2_ environment and the generation of large amounts of SCFAs in the colon, we examined the relationship between SCFAs and HIF stabilization. In the current studies, we identify butyrate as a direct, noncompetitive PHD inhibitor, a testament to the co-evolution of mammals with our commensal microbes. Identification of this new mechanism of PHD inhibition offers therapeutic potential using this endogenous metabolite.

## Results

### Butyrate stabilizes HIF independent of oxygen consumption

**Figure 1.**
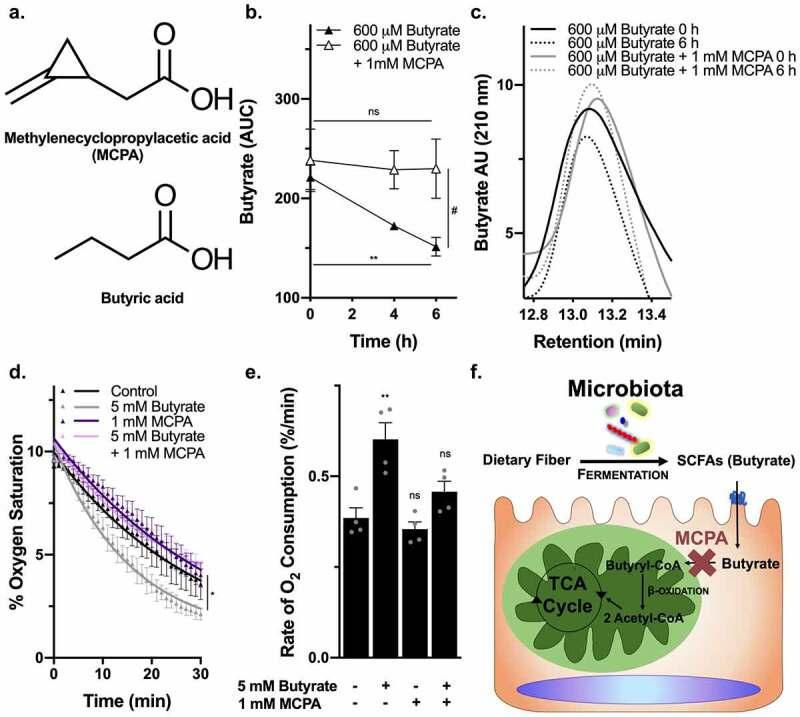
*MCPA inhibits β-oxidation of butyrate to diminish oxygen consumption*. (a) Chemical structure of MCPA compared to butyrate. (b) Relative butyrate concentration remaining in media between 0 to 6 h of either 600 μM butyrate or 600 μM butyrate with 1 mM MCPA treatment in T84 cells ((*n* = 3, error bars: SEM, *ns* not significant, * *p* < .05 by 1-way ANOVA, Fisher’s multiple comparison; *t* = 2.514, df = 4, # *p* < .1 by unpaired two-tailed student’s t-test). (c) High performance liquid chromatography (HPLC) tracings of butyrate at 0 h and 6 h for 600 μM butyrate or 600 μM butyrate with 1 mM MCPA treatment in T84 cells. (d) Oxygen saturation of T84 cells treated with 5 mM butyrate with or without 1 mM MCPA over 30 min (*n* = 3, error bars: SEM, * *p* < .05 by 1-way ANOVA; One phase decay least squares fit). (e) Rates of oxygen consumption calculated from nonlinear regression of oxygen saturation data in T84 cells treated with 5 mM butyrate with or without 1 mM MCPA (*n* = 4, error bars: SEM, *ns* not significant, ** *p* < .01 by 1-way ANOVA, Fisher’s multiple comparison). (f) Illustration of the mechanism of MCPA to inhibit β-oxidation of butyrate

Previously published work from our group showed that the metabolism of butyrate stabilized HIF through a mechanism involving increased oxygen consumption.^12^ However, utilizing the ATP synthase inhibitor oligomycin, it was revealed that oxygen consumption did not fully explain HIF stabilization. Indeed, residual HIF activity was evident in the presence of saturating concentrations of oligomycin^12^ To more fully determine the nature of increased β-oxidation and consequent oxygen consumption by butyrate metabolism, we utilized methylene cyclopropyl acetic acid (MCPA) to irreversibly inhibit SCFA acyl-CoA dehydrogenases, most potently and specifically butyryl-CoA dehydrogenase to block butyrate β-oxidation (Figure 1a).^14^ MCPA has been shown to irreversibly inhibit the metabolism of butyrate and reduce acetyl-CoA levels by 70–90% in rat hepatocytes.^15^ MCPA reduced butyrate metabolism in T84 cells (Figure (1b, c)) and eliminated the increase in oxygen consumption associated with butyrate (Figure (1d, e)). As depicted in figure 1f, MCPA inhibits β-oxidation of butyrate, preventing butyrate-derived acetyl-CoA production and therefore butyrate-induced TCA cycle flux and oxygen consumption through oxidative phosphorylation/aerobic respiration.

**Figure 2.**
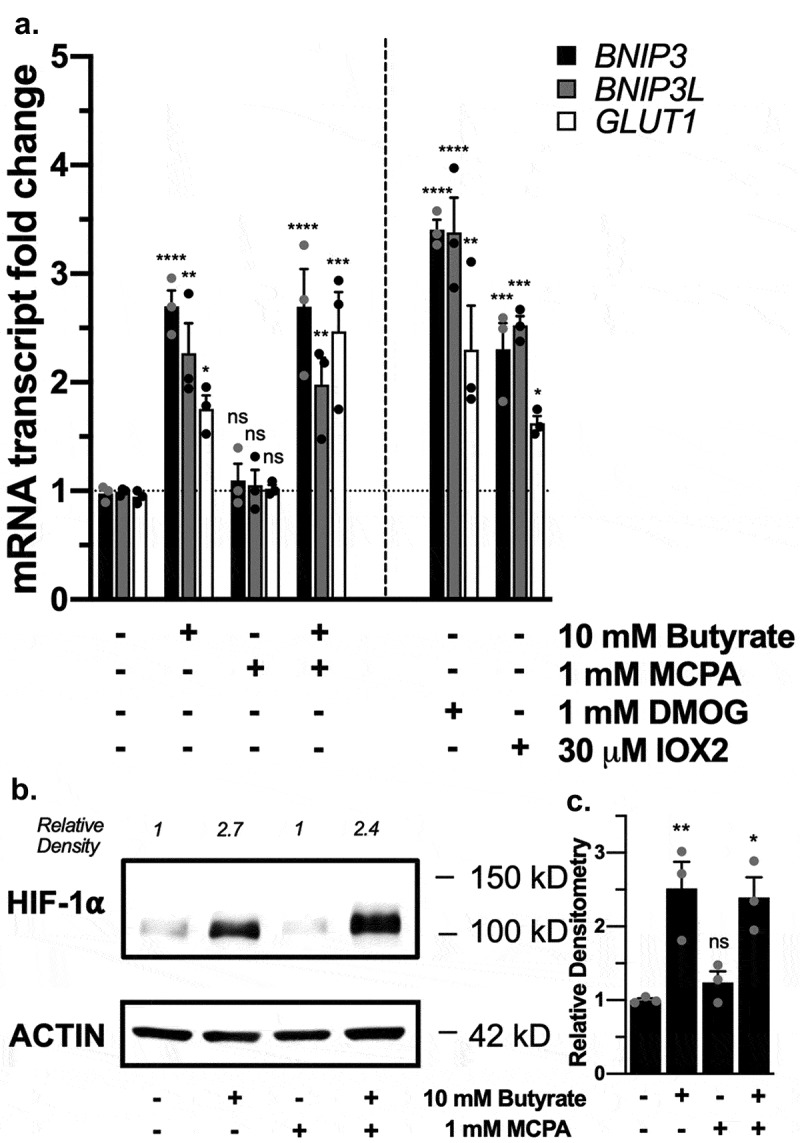
*Butyrate stabilization of HIF in the presence of β-oxidation inhibition*. (a) HIF-1α target mRNA expression in T84 cells treated with 10 mM butyrate with or without 1 mM MCPA, 1 mM DMOG, or 30 μM IOX2 for 4 h (*n* = 3, error bars: SEM, *ns* not significant, * *p* < .05, ** *p* < .01, *** *p* < .001, **** *p* < .0001 by 1-way ANOVA, Fisher’s multiple comparison). (b) HIF-1α protein expression in T84 cells treated with 10 mM butyrate with or without 1 mM MCPA for 4 h. (c) Quantified densitometry of HIF-1α protein expression in T84 cells treated with 10 mM butyrate with or without 1 mM MCPA for 4 h (*n* = 3, error bars: SEM, *ns* not significant, * *p* < .05, ** *p* < .01 by 1-way ANOVA, Fisher’s multiple comparison)

In agreement with our previous work,^12^ we confirmed in T84 adenocarcinoma model IECs the ability of butyrate to stabilize HIF through the induction of HIF-1α target genes *BNIP3, BNIP3L*, and *GLUT1* (Figure 2a), similar to dimethyloxalylglycine (DMOG), a 2-OG analogue with broad-spectrum inhibition of PHDs, and IOX2, a more PHD2-specific inhibitor.^10,12,16^ HIF-1α protein levels were also increased with butyrate (Figure 2(b,c)). In the presence of MCPA, butyrate still stabilized HIF (Figure 2(a,d)); thus, butyrate does not stabilize HIF solely through limiting oxygen availability. We found similar HIF stabilization in A549 lung adenocarcinoma epithelial cells and HMEC-1 human dermal microvascular endothelial cells seen by HIF-1α target gene induction and increased HIF-1α protein levels (Supplemental Figure 1a-c), suggesting a more universal response beyond IECs.

### Butyrate increases 2-OG similar to PHD inhibition

**Figure 3.**
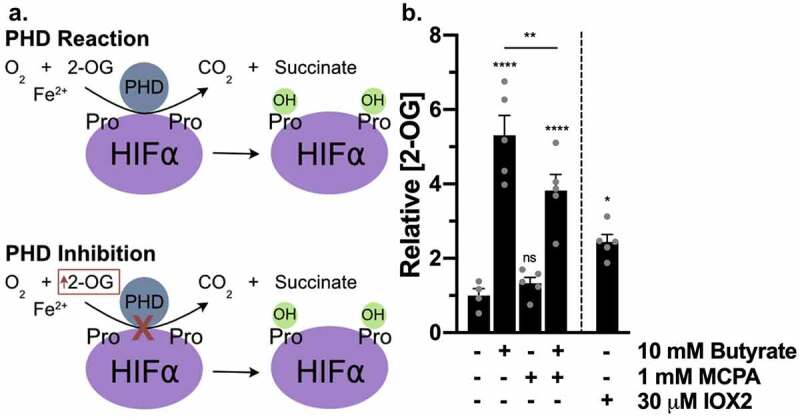
*PHD inhibitors and butyrate increase 2-OG*. (a) PHD enzymatic reaction of O_2_ and 2-OG converted to CO_2_ and succinate in order to hydroxylate HIF-α proline residues. Inhibition of PHD results in increased 2-OG. (b) 2-OG concentrations in T84 cells treated with 10 mM butyrate with or without 1 mM MCPA or 30 μM IOX2 for 3 h (*n* = 4–5, error bars: SEM, *ns* not significant, * *p* < .05, ** *p* < .01, **** *p* < .0001 by 1-way ANOVA, Fisher’s multiple comparison)

To more fully understand the relationship between butyrate and HIF, we examined whether butyrate influences PHD activity by monitoring 2-OG levels. Olenchock et al.^17^ established that PHD2 inhibition directly leads to 2-OG accumulation, as PHD2 decarboxylates 2-OG at a high rate of ~200 pmol/min/g of tissue, with 1 mole of PHD2 estimated to decarboxylate 45 moles of 2-OG in 1 min. In this, we considered such 2-OG accumulation as a metabolic biomarker of PHD inhibition (Figure 3a). Butyrate significantly increased 2-OG levels in T84 IECs compared to control after 3 h, as did the PHD inhibitor IOX2, albeit to lesser extent than butyrate at this time point (Figure 3b). In the presence of MCPA and butyrate, which eliminated the β-oxidation of butyrate, 2-OG levels were also significantly increased, expectedly to a significantly lesser level compared to butyrate alone (Figure 3b). MCPA, through eliminating β-oxidation of butyrate, not only stops the increase in oxygen consumption, but also prevents the increased TCA cycle production of metabolites such as 2-OG (figure 1f). Because 2-OG is also a TCA cycle metabolite, the additional increase in 2-OG with butyrate compared to IOX2 and butyrate with MCPA represents the 2-OG produced from the TCA cycle because of butyrate β-oxidation. Butyrate treatment over 4 days has been shown to significantly increase acetyl-CoA and 2-OG levels in other model IECs.^18^ Our findings here suggest that while butyrate metabolism contributes to increased 2-OG, butyrate additionally increases 2-OG levels in a manner independent of β-oxidation that is possible through direct PHD inhibition.

### Butyrate directly inhibits recombinant PHD2_181-402_

**Figure 4.**
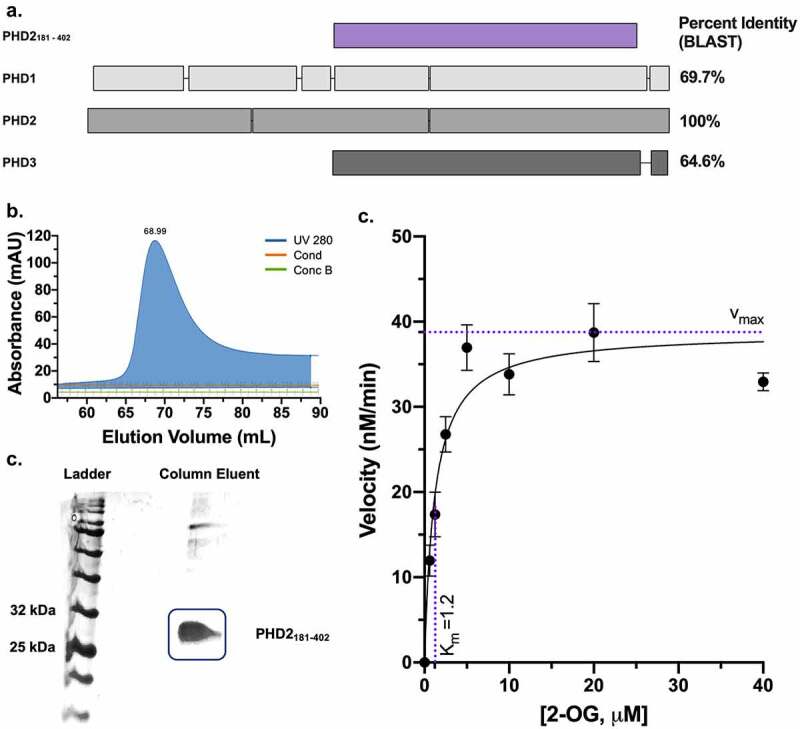
*Recombinant PHD2_181-402_ expression and Michaelis-Menten constant (K_m_) determination of 2-OG for PHD2_181-402_*. (a) PHD2_181-402_ compared to PHD1 (407 amino acid residues), PHD2 (426), and PHD3 (239), showing overall homology between the three human PHDs and PHD2_181-402_ spanning the highly conserved active site. Boxes indicate expressed amino acids, and overlap between boxes indicates amino acid sequence homology. Percent identity (BLAST) is indicated for each PHD isoform in comparison to PHD2_181-402_. (b) Size exclusion superdex 75 column elution of PHD2_181-402_. (Cond: conductivity (mS/cm), concB: percentage of buffer B used). (c) 12% SDS gel image of the Superdex 75 column eluent confirming the expression and purity of PHD2_181-402_. (d) 100 nM of PHD2_181-402_ enzyme was incubated with various concentrations of substrate 2-OG from 625 nM to 40 μM,10 μM HIF-1α_547-581_ peptide, 10 μM Fe (II), and 100 μM ascorbic acid and concentration of product succinate was measured after a 10-minute reaction to determine the rate (*n* = 4, error bars: SEM, Michaelis-Menten least squares fit)

To pinpoint if butyrate inhibits PHDs directly, we expressed recombinant PHD derived from the human PHD2 sequence. There are three human PHDs: PHD1, PHD2, and PHD3, with PHD2 being the most abundant and expressed in the majority of tissues, the most important regulator of HIF, particularly HIF-1α, and the only PHD that is embryonically lethal when deleted.^19^ PHD1 is exclusively localized to the nucleus, whereas PHD2 is mainly cytoplasmic, and PHD3 is both.^11^ We did not attempt to use full-sized PHD2, as it is problematic to express and stabilize, conferring truncated versions of the protein that do not influence catalytic activity as the field standards for assays.^20^ While PHD2_181-426_ is the commonly utilized catalytic domain, we expressed PHD2_181-402_, which is similar in activity.^21^ Sequence comparisons and modeling studies indicate that the PHD2 active site is highly conserved among the PHDs.^22^ We posit that the results garnered from the recombinant PHD2 are likely applicable to all PHDs, as PHD2_181-402_ spans the conserved active site while excluding specificity determining regions in the N-terminal domains (Figure 4a).^23^ While PHD2_181-402_ does contain the non-homologous β2β3 loop within the catalytic domain that determines the preference of PHD3 for the C-terminal ODD (CODD) of HIF-1α, the HIF-1α peptide utilized in our assays only contains the CODD and mitigates such specificity differences. Recombinant PHD2 was purified prior to use on size-exclusion column Superdex 75, and the expected size of 28.9 kDa was verified on SDS gel electrophoresis (Figure (4b, c)).

We next evaluated the activity of PHD2_181-402_ using a bioluminescent succinate detection assay as described by Alves *et al*.^24^ PHD2_181-402_ was confirmed to be catalytically active with an optimal protein concentration for the assay of 1 μM (Supplemental Figure 2), in agreement with literature and allowing the reaction to remain within the limits of detection for the assay.^24^ The K_m_ of 2-OG for PHD2_181-402_ was determined as 1.2 μM (Figure 4d), in agreement with literature.^25^

**Figure 5.**
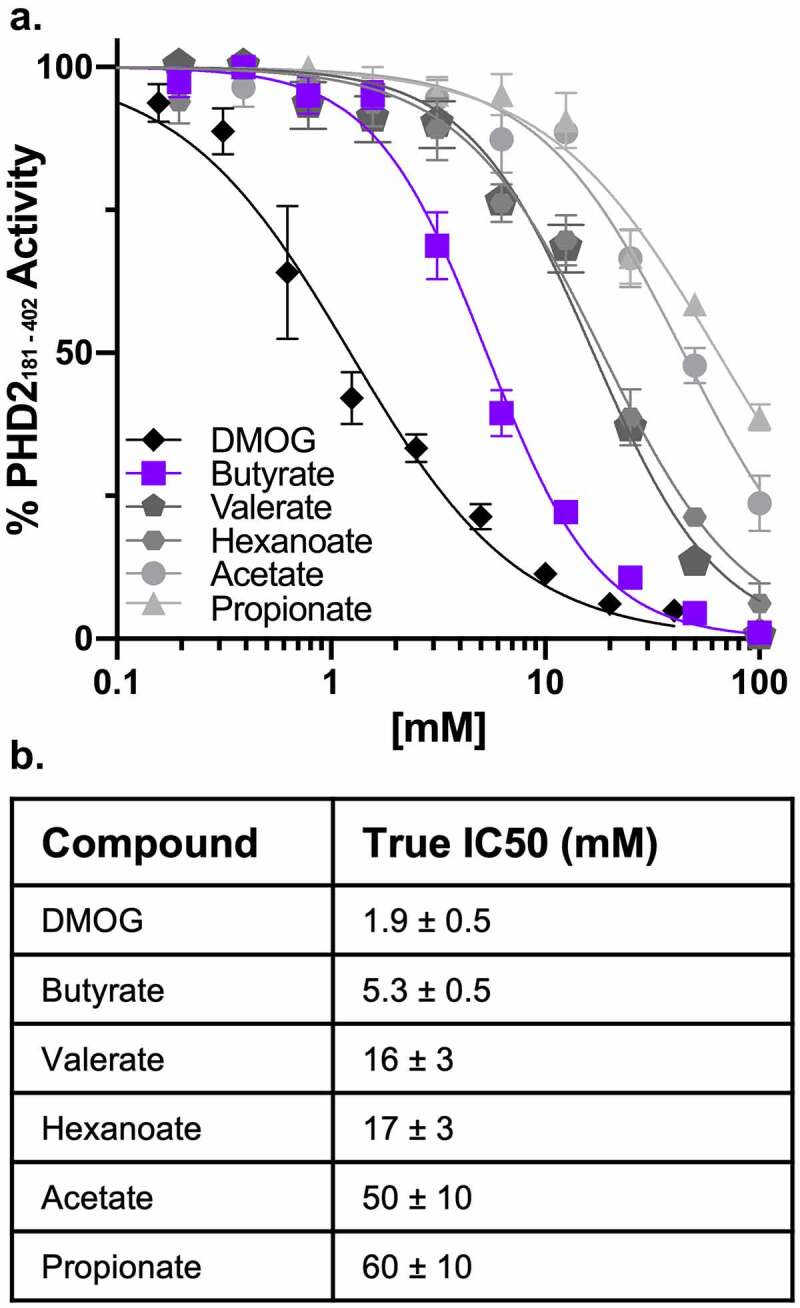
*Butyrate specifically and noncompetitively inhibits PHD2_181-402_*. (a) Representative percentage of PHD2_181-402_ activity normalized to control based on succinate production following a 10-minute reaction of 1 μM PHD2_181-402_, 10 μM 2-OG, 10 μM Fe (II), 10 μM HIF-1α_547-581_ peptide (CODD), and 100 μM ascorbic acid in the presence of 195.3 μM to 100 mM SCFAs (acetate, propionate, butyrate, valerate, and hexanoate) and 78 μM to 40 mM DMOG (*n* = 4, error bars: SEM, [Inhibitor] vs. normalized response – variable slope least squares fit). (b) True IC50 values for SCFAs and DMOG calculated from measured IC50 values accounting for percentage of substrate conversions between replicate experiments (*n* = 5–12, error: SEM)

We found that butyrate most potently inhibited PHD2_181-402_ compared to all other tested SCFAs, with a true half maximal inhibitory concentration (IC50) of 5.3 ± 0.5 mM (Figure (5a, b)). Shorter SCFAs acetate and propionate were ~10-fold less effective at inhibiting PHD2_181-402_, and longer SCFAs valerate and hexanoate were ~3-fold less effective. For the inhibition studies, we calculated true IC50s from measured IC50s with the equation derived by Wu *et al*.^26^ describing the relationship between the measured IC50 of an inhibitor and the percentage of substrate conversion, as Michaelis–Menten kinetics requires substrate conversion to be below 10%, which would produce signals difficult to detect with our assay.^26^ As a positive control in support of the assay, we determined the true IC50 for DMOG to be 1.9 ± 0.5 mM, within the range of reported IC50s between 2.89 μM to 4 mM and is broad due to the inherent dependency of IC50s on assay conditions that varies across studies.^16,25,27^ Thus, butyrate is comparable to DMOG as a PHD inhibitor.

### Butyrate binds directly and specifically to PHD2_181-402_

Next, we established whether butyrate binds directly to PHD2_181-402_ using 1D WaterLOGSY NMR.^28^ This method enables sensitive and robust detection of binding (dissociation constants as weak as μM to low mM) based on the magnetization transfer between water molecules, proteins, and ligands of interest in close proximity via dipolar proton–proton cross-relaxation (nuclear Overhauser effect, NOE). Protein-ligand complexes exhibit an opposing NOE with water, resulting in a positive WaterLOGSY signal, while molecules that do not bind to protein exhibit a weak and same NOE with water, resulting in a negative WaterLOGSY signal. Binding and non-binding ligands can be distinguished in a WaterLOGSY spectrum via their opposite signs in relation to water for their corresponding peaks.

**Figure 6.**
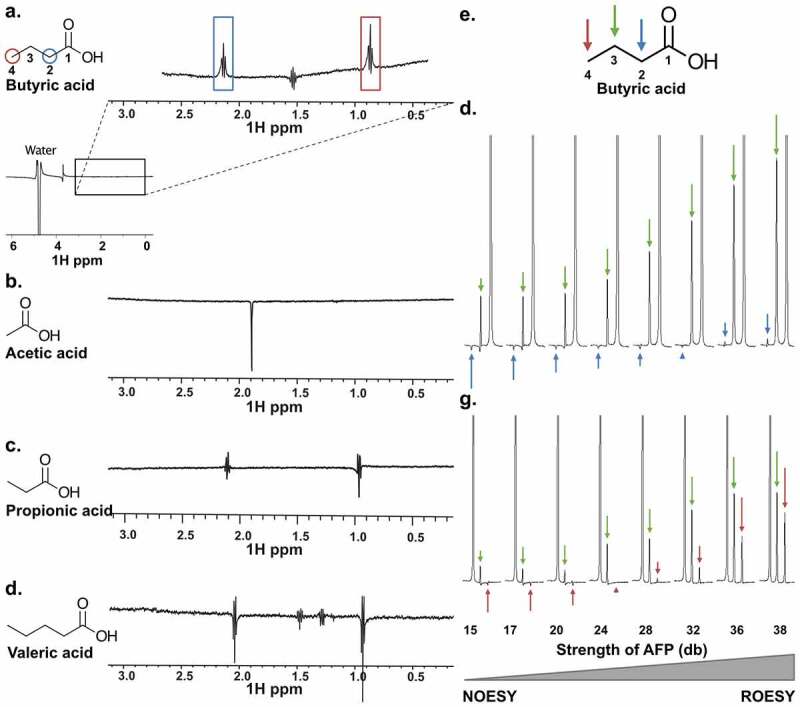
*NMR characterization of SCFAs binding to PHD2_181-402_*. (a-d) WaterLOGSY 1D NMR to determine SCFAs binding to PHD2_181-402_. 10 mM of each tested SCFA was mixed with 25 μM PHD2_181-402_. ((a) Butyric acid; peak inversions relative to the water signal were seen for protons directly bound to carbons C2 and C4, indicating binding. (b-d) Acetic acid, propionic acid and valeric acid, respectively; no peak is inverted, indicating no binding under these experimental conditions. (e-g) AFP-NOESY 1D NMR to determine butyrate atoms in the binding pocket of PHD2_181-402_. (e) The butyrate structure with labeled positions and corresponding arrow color code. (f) 10 mM butyrate was mixed with 25 μM PHD2_181-402_. The strength of the adiabatic pulse was gradually increased to shift relaxation contributions from longitudinal cross relaxation (NOESY) to transverse cross relaxation (ROESY). Protons attached to C4 were selectively inverted and acted as source of magnetization transfer. Peaks of protons attached to C2, unlike protons attached to C3, showed a profile typical for strong spin diffusion. This indicates embedding of the C2 in the binding pocket of the protein. (g) Protons attached to C2 were selectively inverted. Peaks of protons attached to C4, unlike protons attached to C3, showed a profile typical for strong spin diffusion, indicating embedding of the C4 protons in the binding pocket. The weak spin diffusion dependence of the protons attached to C3 in both (F) and (G) indicates that the C3 protons are more solvent exposed

The combination of PHD2_181-402_ and butyrate revealed two inverted proton peaks at 2.02 and 0.75 ppm compared to the water signal and demonstrate a clear positive signal, indicative of binding (Figure 6a). By contrast, we did not observe binding of other SCFAs under similar conditions (Figure (6b, d)), as these peaks were weak and negative, suggesting that SCFA binding to PHD is specific for butyrate. We further validated the specific butyrate protons involved in the butyrate-PHD2_181-402_ interaction through 1D AFP-NOESY NMR (Figure 6e), in which a linear combination of two different proton–proton cross-relaxation rates (NOEs and rotating-frame Overhauser effects, ROEs) are measured during adiabatic fast passage (AFP). This technique allows for mapping of the ligand pharmacophore by monitoring the response of individual protons to an increase of ROEs contribution to the overall cross-relaxation rate.^29^ The protons resonating at 2.02 (figure 6f) and 0.75 ppm (Figure 6g) experienced “spin diffusion” modification, indicative of deeper embedding in the binding pocket, while the proton resonating at 1.41 ppm did not exhibit such a trend (figure (6f, g)). These experiments together established that the protons bound to carbons C2 (2.02 ppm) and C4 (0.75 ppm) of butyrate directly interact with PHD2_181-402_, while the protons attached to C3 (1.41 ppm) appear to be more solvent exposed. In agreement with these results, any C2 or C4 modification resulted in significant decreases in inhibition by increasing IC50s, whereas modifications to C3 decreased inhibition to a lesser extent (Table 1). Together, these results confer butyrate as a highly specific PHD_181-402_ inhibitor compared to other SCFAs.

**Table 1.** *True IC50 values of butyrate-derived compounds with modifications on different carbons*. Modifications to C2 and C4 of butyrate significantly decrease inhibition of compounds for PHD2_181-402_, while modifications to C3 impact inhibition to a lesser extent (*n* = 4, error: SEM)

Compound	Structure	True IC50 (mM)
Butyrate		5.3 ± 0.5
2-Ethylbutyrate		77 ± 6
2-Methylbutyrate		77.2 ± 0.3
3-Hydroxybutyrate		14 ± 1
4-Acetylbutyrate		220 ± 50

### Butyrate noncompetitively inhibits PHD2_181-402_ with a K_i_ of 5.3 mm

**Figure 7.**
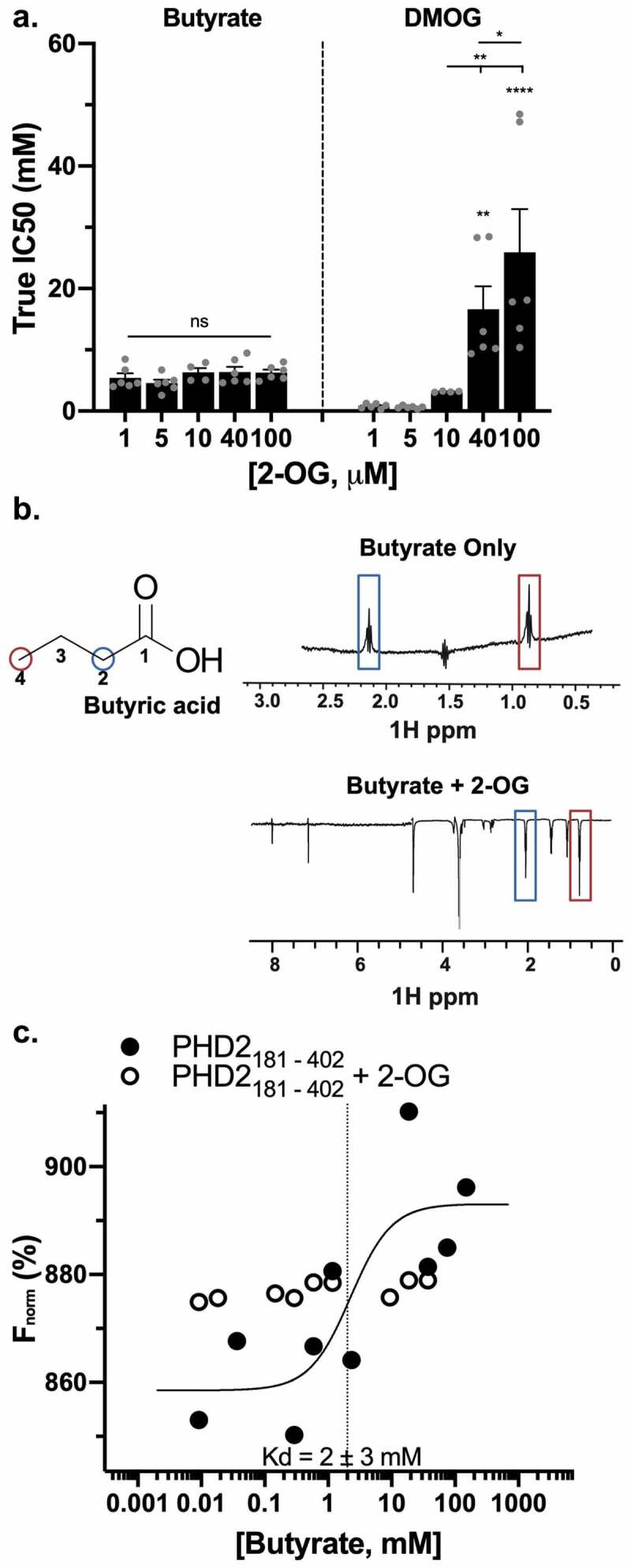
*Butyrate noncompetitively inhibits PHD2_181-402_*. (a) True IC50 values for butyrate and DMOG in the presence of increasing 2-OG concentrations (*n* = 4–6, error bars: SEM, *ns* not significant, * *p* < .05, ** *p* < .01, **** *p* < .0001 by 1-way ANOVA, Fisher’s multiple comparison). (b) WaterLOGSY 1D NMR of 10 mM butyrate incubated with 25 μM PHD2_181-402_ with and without 150 μM 2-OG. No peak inversion relative to the water signal was observed for any of the butyrate resonances indicating absence of binding in the presence of 2-OG. (c) Representative MST plot of 25 nM PHD2_181-402_ incubated with a range of butyrate concentrations from 9.2 μM to 75 mM with or without 500 nM 2-OG and assayed for protein and ligand interaction to determine binding affinity of butyrate to PHD2_181-402_. Measurements were repeated 3 times

By titrating different concentrations of substrate 2-OG into the inhibition assay and assessing if IC50s increased, we found the mode of butyrate inhibition to be either noncompetitive or uncompetitive. IC50s for butyrate did not change with increasing 2-OG (Figure 7a), whereas IC50s for DMOG increased significantly with increasing 2-OG, in agreement with DMOG as a known competitive 2-OG analogue.^16,24^ Uncompetitive inhibitors only bind to the enzyme substrate complex, whereas noncompetitive inhibitors can bind to enzyme alone or enzyme substrate complex since the inhibitor has a unique binding site distinct from the substrate-binding site.^27^ We found that butyrate could not bind to the 2-OG and PHD2_181-402_ complex, as the inverted peaks at 2.02 and 0.75 ppm were lost on WaterLOGSY 1D NMR (Figure 7b), eliminating uncompetitive inhibition as a binding mechanism. We further confirmed butyrate binding to PHD2_181-402_ using microscale thermophoresis (MST), which revealed a dissociation constant (K_D_) for butyrate of 2 ± 3 mM (Figure 7c), comparable to our true IC50 for butyrate. Again, in the presence of 2-OG, MST revealed no butyrate binding to PHD2_181-402_ (Figure 7c). In the context of these assays, the lack of butyrate binding to PHD2_181-402_ in the presence of 2-OG does not represent competitive inhibition, as we already ruled out competitive inhibition with the data shown in Figure 7a, suggesting that butyrate binds PHD2_181-402_ elsewhere of the 2-OG binding site. Allosteric regulation could result in the formation of the 2-OG and PHD2_181-402_ complex causing a conformational change in PHD2_181-402_ that precludes butyrate binding, similar to the way a noncompetitive inhibitor-like butyrate could bind to PHD2_181-402_ and cause a conformational change that precludes substrates like 2-OG from binding. Thus, our findings indicate that butyrate functions as a noncompetitive PHD inhibitor with a unique binding site.

IC50s depend on exact experimental conditions, making direct comparisons and global applications difficult. We utilized the IC50 to K_i_ conversion equation detailed by Cer *et al*.^27^ to determine the intrinsic inhibitory constant (K_i_) of butyrate for PHD2_181-402_ that is independent of experimental variables. We calculated the noncompetitive K_i_ of butyrate for PHD2_181-402_ to be 5.3 mM, a physiologically relevant concentration for butyrate *in vivo*.

### Microbiota-derived butyrate is essential to stabilizing colonic HIF

**Figure 8.**
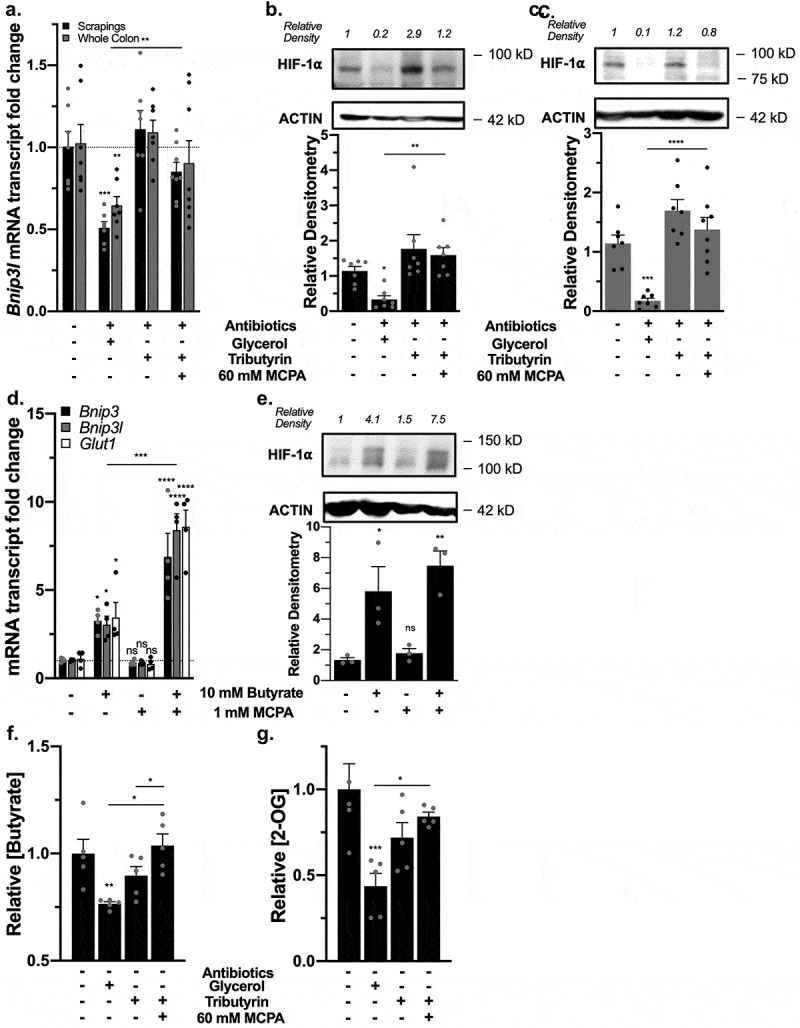
*Microbiota-derived butyrate inhibits PHDs in vivo*. (a) HIF-1α target mRNA expression of *Bnip3l* in murine whole colon tissue and intestinal epithelial scrapings of control mice, mice treated with antibiotics (glycerol control), and mice given back tributyrin (200 µL, 3 d) with or without 60 mM MCPA for 4 h (*n* = 7, error bars: SEM, * *p* < .05, ** *p* < .01, *** *p* < .001 by 1-way ANOVA, Fisher’s multiple comparison). (b) HIF-1α protein expression in murine intestinal epithelial scrapings with corresponding quantified densitometry in mice treatment groups (*n* = 7, error bars: SEM, * *p* < .05, ** *p* < .01 by 1-way ANOVA, Fisher’s multiple comparison). (c) HIF-1α protein expression in murine whole colon tissue with corresponding quantified densitometry in mice treatment groups (*n* = 7, error bars: SEM, *** *p* < .001, **** *p* < .0001 by 1-way ANOVA, Fisher’s multiple comparison). (d) HIF-1α target mRNA expression in murine enteroids differentiated of epithelial lineage treated with 10 mM butyrate with or without 1 mM MCPA for 4 h (*n* = 4, error bars: SEM, * *p* < .05, *** *p* < .001, **** *p* < .0001 by 1-way ANOVA, Fisher’s multiple comparison). (e) HIF-1α protein expression in murine enteroids treated with 10 mM butyrate with or without 1 mM MCPA for 4 h with corresponding quantified densitometry (*n* = 4, error bars: SEM, * *p* < .05, ** *p* < .01 by 1-way ANOVA, Fisher’s multiple comparison). (f) Butyrate levels in murine whole colon tissue of mice treatment groups (*n* = 7, error bars: SEM, * *p* < .05, ** *p* < .01 by 1-way ANOVA, Fisher’s multiple comparison). (g) 2-OG levels in murine whole colon tissue of mice treatment groups (*n* = 7, error bars: SEM, * *p* < .05, *** *p* < .001 by 1-way ANOVA, Fisher’s multiple comparison)

We next confirmed that butyrate functions as a PHD inhibitor *in vivo* through antibiotic-mediated depletion of the gut microbiota and SCFAs in mice. This approach has been previously published and validated by our group and was shown to deplete all SCFA by >90%.^4,12^ As we previously published, we administered tributyrin (200 µL), a prodrug of butyrate composed of a glycerol backbone with three butyrate moieties, to SCFA-depleted mice via oral gavage every day for 3 days with glycerol as a control. Additionally, some mice given tributyrin were subjected with a rectal gavage of 60 mM MCPA to inhibit butyrate metabolism 4 h prior to sacrifice. Previous studies have found that oral gavage with tributyrin elevates plasma butyrate concentrations to > 1 mM 1 h after dosing in mice.^30^ 60 mM MCPA was chosen as a well-tolerated dose that inhibited β-oxidation in mice and rats.^31^ This analysis revealed in both whole-colon tissue and intestinal epithelial scrapings from these mice that antibiotic treatment decreased HIF stabilization as HIF-1α target gene *Bnip3l* was significantly decreased that was normalized by both tributyrin supplementation with or without MCPA (Figure 8a). HIF-1α protein was also diminished in epithelial scrapings (Figure 8b) and whole-colon tissue (Figure 8c) with antibiotic treatment, which was also rescued with tributyrin supplementation with or without MCPA. Additionally, we confirmed in differentiated murine enteroids that butyrate with or without MCPA treatment induced expression of HIF-1α target genes (Figure 8d), as well as increased HIF-1α protein expression after 4 h (Figure 8e). These results, in conjunction with the colon epithelial scrapings, confirm that PHD inhibition does occur in epithelial cells in a similar manner as our *in vitro* experiments in T84 IECs revealed. However, the stabilization of HIF-1α in whole-colon tissue also is in agreement with our findings in HMEC-1 and A549 cells that PHD inhibition by butyrate could expand to beyond just IECs alone. We confirmed that colon tissue butyrate levels were significantly decreased with antibiotic treatment (glycerol control) and were then rescued by tributyrin with or without MCPA treatment (figure 8f). As MCPA is a butyrate metabolism inhibitor, the MCPA rectal gavage in mice given tributyrin significantly increased the level of butyrate in the colon tissue compared to just tributyrin alone. Lastly, antibiotic treatment depleted 2-OG levels in the mice colon tissues that was then recovered by tributyrin treatment with or without MCPA (Figure 8g). Our results suggest that microbiota-derived butyrate also stabilizes HIF through direct PHD inhibition, as indicated by HIF stabilization, induction of HIF-1α mRNA targets, and a 2-OG increase even during MCPA inhibition of butyrate metabolism (TCA cycle) and accompanying oxygen consumption.

To confirm these results, we reconstituted butyrate in a second manner in SCFA-depleted mice. These mice were rectally administered 100 mM butyrate with or without 10 mM MCPA or 1.5 mM of the PHD2 inhibitor IOX2 acutely for 1 h. Daily 100 mM butyrate enemas over weeks have been used therapeutically in both rat and mice animal studies and human clinical trials.^32–34^ Doses for MCPA and IOX2 were selected to be optimized for their influences as well as be tolerated in the rapid 1 h time point, which was selected as Olenchock *et al*^17^ demonstrated that 2-OG levels increased in liver tissues after pharmacological PHD inhibition to a peak within 10 minutes and decreased to nonsignificant levels after 4 h. Furthermore, in addition to the possibility that butyrate could inhibit PHDs and elicit measurable responses in a rapid manner, we targeted 1 h to decrease the likelihood of seeing metabolic shifts due to butyrate metabolism in addition to PHD inhibition alone, similar to our T84 *in vitro* studies.

**Figure 9.**
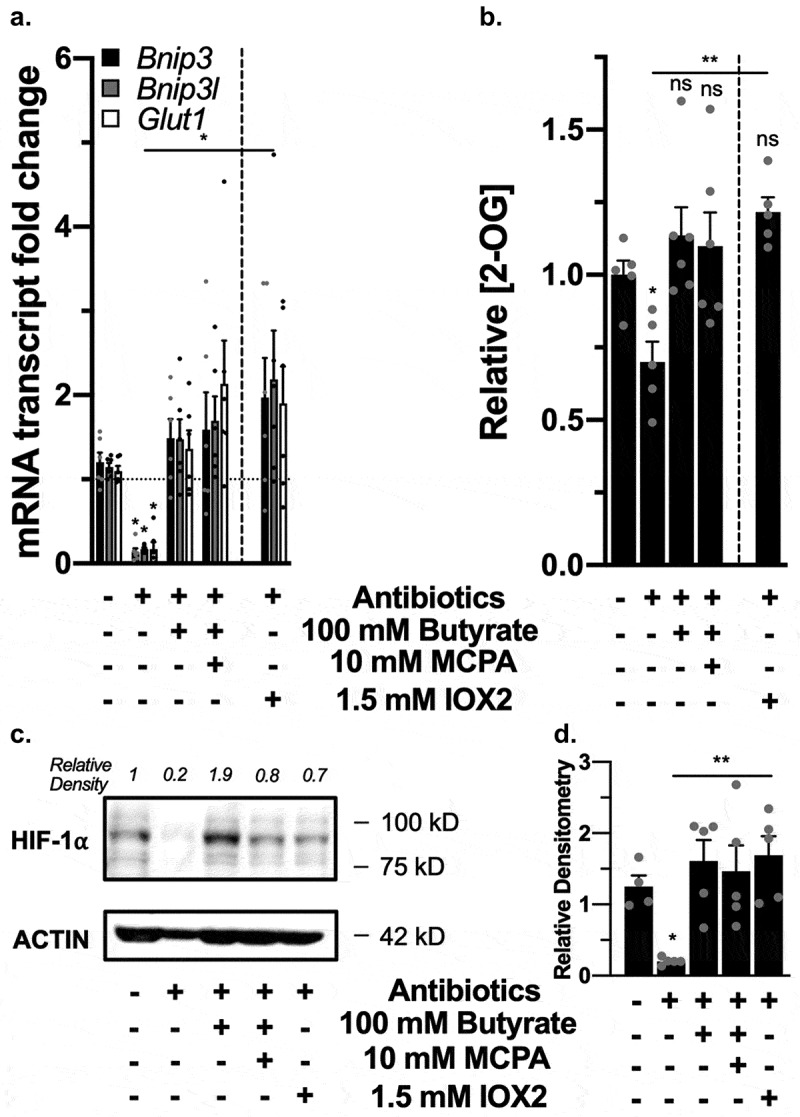
*Rectal gavage of butyrate inhibits PHDs in vivo*. (a) HIF-1α target mRNA expression in murine colon tissue of control mice, mice treated with antibiotics for microbiota and microbial-derived butyrate depletion, and mice rectally given back 100 mM butyrate, 100 mM butyrate with 10 mM MCPA, and 1.5 mM IOX2 for 1 h (*n* = 6, error bars: SEM, * *p* < .05, ** *p* < .01 by 1-way ANOVA, Fisher’s multiple comparison). (b) 2-OG levels in murine colon tissue following antibiotics and rectal gavage treatments (*n* = 5–6, error bars: SEM, *ns* not significant, * *p* < .05, ** *p* < .01 by 1-way ANOVA, Fisher’s multiple comparison). (c) HIF-1α protein expression in murine colon tissue. (d) Quantified densitometry of HIF-1α protein expression in murine colon tissue treated with rectal gavage of 100 mM butyrate with or without 10 mM MCPA or 1.5 mM IOX2 for 1 h (*n* = 4, error bars: SEM, *ns* not significant, * *p* < .05, ** *p* < .01 by 1-way ANOVA, Fisher’s multiple comparison)

Antibiotics significantly decreased mRNA expression of HIF-1α targets *Bnip3, Bnip3l*, and *Glut1* that were rescued with butyrate, butyrate with MCPA, and IOX2 (Figure 9a). Similarly, using colon tissue 2-OG levels as a biomarker for PHD inhibition, we observed a significant decrease in 2-OG levels with antibiotics that were rescued with both butyrate and IOX-2 (Figure 9b). Lastly, western blot analysis revealed the loss of HIF-1α protein stabilization with antibiotics that returned with all treatments (Figure (9c, d)), in support of our hypothesis that butyrate functions as a direct PHD inhibitor *in vivo*.

## Discussion

Microbiota-derived butyrate is critical to maintaining intestinal homeostasis, and dysbiosis of the microbiota in disease states commonly diminishes butyrate levels through decreasing butyrate-producing bacteria, notably in inflammatory bowel diseases (IBD).^35,36^ IBD colonocytes do not effectively transport nor metabolize butyrate, and germ-free mice lacking in butyrate show diminished oxidative metabolism and energy deficiency.^37–39^ Butyrate inhibition of PHDs stabilizes HIF-1⍺, which regulates many critical gut homeostasis genes including claudin 1 (CLDN1), an essential tight junction protein, mucin 2 (MUC2), the major component of the mucus layer, and human beta defensin-1 (DEFB1), an antimicrobial peptide.^40,41^

IECs exist in a state of perpetual low oxygen tension, a phenomenon termed “physiologic hypoxia.”^8^ This is partially due to proximity to the anaerobic colonic lumen, which establishes a radial oxygen gradient with the intestinal epithelium residing at a pO_2_ of less than 10 mmHg or ~1% oxygen to ~5-10% in the vascularized submucosa and muscle layers, but also results from the consumption of oxygen stemming from the metabolism of microbiota-derived butyrate.^12,42^ Germ-free and antibiotic-treated mice show diminished physiologic hypoxia, secondary to lacking intact gut microbiotas and butyrate.^12^ In this the colonic mucosa contributes to an environment with low oxygen levels, in which HIF is stabilized at baseline. Our findings that butyrate directly inhibits PHDs to stabilize HIF could present an even more precise regulation of HIF by the microbiota. As the ETC can function at near anoxia and only becomes limited by intracellular oxygen when levels reach below 0.3%,^43,44^ temporally, butyrate binding to and inhibiting PHDs to stabilize HIF would occur before oxygen becomes limiting from butyrate metabolism. Not only does additional HIF stabilization beyond the oxygen-regulated baseline confer homeostatic benefit, but this rapid stabilization of HIF could also play a major role in priming the colon tissue toward butyrate metabolism in the already oxygen-deprived environment. HIF-1α has been to show to upregulate pyruvate dehydrogenase kinases (PDKs), PDK1 and PDK3, which inactivate the pyruvate dehydrogenase complex (PDC) by inhibiting pyruvate dehydrogenase (PDH) to prevent glucose-derived pyruvate conversion into acetyl-CoA and entering the TCA cycle, and thus shifts the production of acetyl-CoA to be from β-oxidation of butyrate.^45,46^ Butyrate has also been shown to strongly induce PDK1-4 through HDAC inhibition.^47^ Ultimately, in the unique environment of the colon, butyrate directly and indirectly through HIF-1α stabilization induces PDKs to shift acetyl-CoA production from glycolysis to butyrate oxidation, cementing the importance and specificity of butyrate to the colon. Our findings further establish microbiota-derived butyrate as an essential component of intestinal homeostasis.

Butyrate inhibition of PHDs also influences intestinal homeostasis through regulating metabolite levels, specifically 2-OG. Increased 2-OG from PHD inhibition drives production of kynurenine, which protects against cardiac ischemia.^17^ Kynurenine, as a tryptophan derivative, promotes intestinal wound healing and alleviates murine colitis,^48^ but can be a downstream beneficial influence of accumulated 2-OG from butyrate PHD inhibition. 2-OG inhibits colorectal carcinogenesis, and 2-OG supplementation downregulates inflammatory cytokines IL-6, IL-22, TNF-α, and IL-1β along with decreasing opportunistic pathogens and increasing mutualistic bacteria including the butyrate-producing class *Clostridia* in the colon.^18,49^ Again, due to the temporal differences in 2-OG levels due to the route of production, whether rapidly from direct inhibition of PHDs or more delayed from β-oxidation, butyrate exhibits meticulous control over the intestinal mucosa. As observed with DMOG and IOX2 treatment in T84 cells, PHD inhibition alone raised 2-OG to a certain level, whereas butyrate demonstrated the capacity to further elevate 2-OG due to both metabolism and PHD inhibition. This highlights an important therapeutic consideration in that PHD inhibition and the contribution of butyrate β-oxidation to the TCA cycle and 2-OG production are co-foundational components of gut homeostasis and may both be necessary for wound healing.

Interestingly, the microbiota produces butyrate to reach levels of 15–25 mM in the colon, and due to differential apical and basolateral affinities of the SCFA-HCO3^−^ exchange transporters, >95% is absorbed and sequestered to the colonic mucosa for signaling and metabolism, with only low micromolar concentration (<2% of colon-derived) found in portal blood, and the remaining secreted in feces.^3,50–52^ However, most acetate and propionate are delivered to and utilized by the liver, with acetate being the only SCFA to enter peripheral circulation at high enough concentrations to additionally influence heart, adipose, kidney, and muscle activity.^3,53,54^ Thus, it is likely that butyrate selectively inhibits PHDs in the colon, while other SCFAs do not reach sufficient levels for such inhibition. It is also notable that such selectivity of butyrate in the colon is not unexpected, as butyrate also most potently inhibits HDACs, while propionate, valerate, and hexanoate exhibit lesser degrees of HDAC inhibition, and acetate shows none.^55,56^

Significant interest lies in developing small-molecule inhibitors of PHDs to stabilize HIF in the treatment of numerous disorders.^57^ Roxadustat became the first PHD inhibitor to pass phase III clinical trials for treatment of renal anemia and has been approved in China and Japan with expected U.S. and global approval soon.^58^ Butyrate, as a well-tolerated, endogenous metabolite, holds potential therapeutic opportunities with few deleterious side effects, especially as a noncompetitive inhibitor. This is a new concept, as developed PHD inhibitors have only focused on targeting the active site. Other iron-chelator classes of inhibitors are designated “noncompetitive” but function to limit iron availability in the active site. Other endogenous inhibitors (e.g. TCA cycle succinate) are considered competitive inhibitors.^10,59^ A truly noncompetitive inhibitor with a unique binding site has yet to be pursued until our findings here.

**Figure 10.**
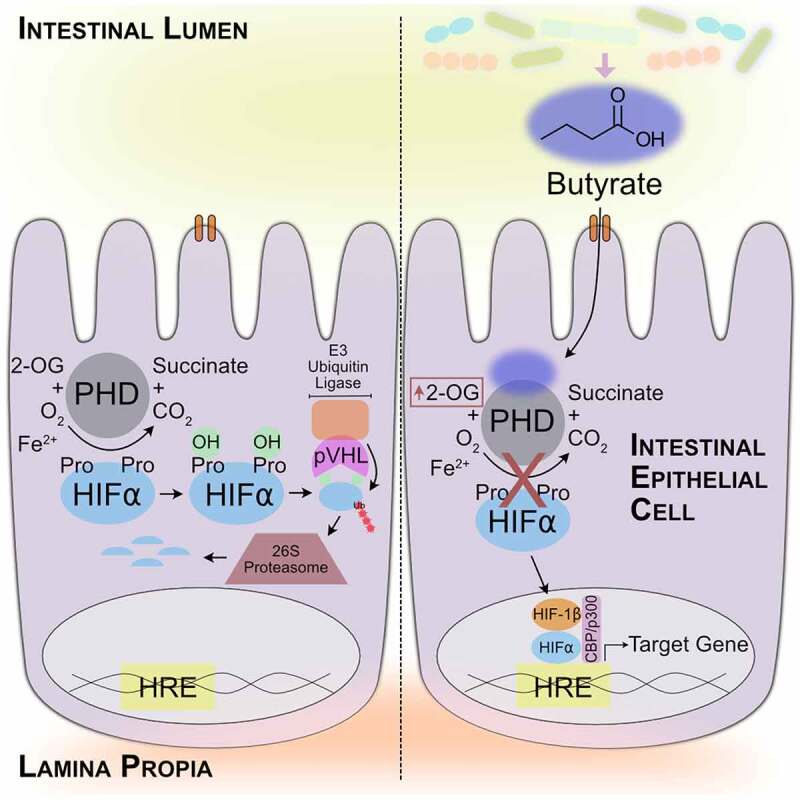
*Microbiota-derived butyrate directly and noncompetitively inhibits PHDs to influence intestinal homeostasis*. Butyrate regulates gene expression through HIF stabilization and impacts metabolite flux through elevating 2-OG levels

Co-evolution of mammals and the gut microbiota has created a complex system in which microbiota-derived butyrate is made available to inhibit PHDs at a specific site and in a tissue-specific manner. However, the introduction of butyrate to other systems could be beneficial in inhibiting PHDs and stabilizing HIF. Our *in vitro* work indicates that butyrate can stabilize HIF in non-intestinal cells and suggests that while organs outside of the colon do not normally experience high concentrations of butyrate, the SCFA may still significantly influence their function. The varied magnitude of HIF-1α gene target responses to butyrate and butyrate with MCPA in A549 cells compared to T84 cells confirm that HIF differentially regulates genes across organ systems, and that butyrate is uniquely metabolized by each organ and exerts distinct influences. Our *in vivo* work also demonstrates that PHD inhibition by butyrate extends beyond just IECs. Overall, our work here demonstrates that microbiota-derived butyrate binds and noncompetitively inhibits PHDs (Figure 10). Such inhibition stabilizes HIF and increases 2-OG, all of which contribute to gut homeostasis. Butyrate as a noncompetitive PHD inhibitor not only provides insight into PHD/HIF therapeutics but also highlights the intricate mutualistic symbiosis that exists between mammals and the microbiota.

## Methods

### Cell culture

T84 cells were cultured in 95% air with 5% CO2 at 37°C in standard media made of DMEM:F-12 supplemented with 10% calf serum, 1% penicillin/streptomycin, and 1% GlutaMAX™ (ThermoFisher Scientific) on 9.6 cm^2^ 6-well plates (ThermoFisher Scientific). Real-time oxygen sensing was done using the SensorDish Reader from Applikon Biotechnology as described previously with T84 cells plated on 0.33 cm^2^ transwell inserts (Corning, 0.4 μm).^12^ All MCPA treatments included a 2 h pretreatment with MCPA before treatment with butyrate.

### Enteroid culture

Murine intestinal organoids/enteroids were isolated from wild-type C57BL/6 mice and cultured as previously described.^60^ Briefly, colonic tissues were minced and then enzymatically digested and dissociated in GentleMACS tubes (Milteny Biotec). The homogenized tissues were then filtered through a 70 µm cell strainer and resuspended in Matrigel (Corning). Cells were cultured in L-WRN conditioned media until ready for differentiation into an epithelial lineage, in which the cells were incubated for 2 d in L-WRN media diluted 5-fold with DMEM:F-12 supplemented with 10% calf serum, 1% penicillin/streptomycin, and 1% GlutaMAX™. Epithelial differentiated enteroids were treated with 5 mM butyrate with or without 1 mM MCPA for 4 h and were analyzed for RNA and protein expression. MCPA treatment also includes a 2 h pretreatment with MCPA before butyrate was added.

### Mouse studies

C57BL/6 mice were purchased from Jackson Laboratories. Animals were maintained and bred in standard housing conditions under 24 h/day, 7 days/week veterinary care available at the University of Colorado Anschutz Medical Campus (AMC) animal facility. All animal procedures were reviewed and approved by the Institutional Animal Care and Use Committee at AMC. 8 to 10-week-old male and female mice were pre-administered an antibiotic cocktail consisting of 1 mg/mL ampicillin, gentamicin, and neomycin, 0.5 mg/mL vancomycin, and 0.25 mg/mL metronidazole for 5 d *ad libitum* in addition to water control mice. Mice were then given 200 µL of tributyrin daily for three days using reusable feeding needles (18060–20, Fine Science Tools). On the third day, 4 h prior to sacrifice, select mice given tributyrin were rectally given 100 μL of 60 mM MCPA with plastic gavage tubes (FTP-20-38-50, Instech) inserted 1 cm into the rectum without surgical lubricant. Whole colon tissue at the site of the rectal gavage was collected for metabolite analysis, while colon whole tissue and epithelial scrapings upstream from the gavage site was collected for RNA and protein analysis. In the second butyrate reconstitution experiment, mice were rectally given 100 μL of 100 mM butyrate, 100 mM butyrate with 10 mM MCPA, 1.5 mM IOX2, or PBS control under anesthesia. Mice were then sacrificed 1 h after rectal supplementation and colon tissues collected for metabolite, mRNA, and protein analysis from the site of the rectal gavage.

### Quantitative PCR

TRIzol® reagent (ThermoFisher Scientific) was used to isolate total RNA from T84 cells. Mouse colon tissue samples were harvested into RNA*later*™ (Invitrogen) and stored at 4°C for 48 h. Total RNA was isolated from the tissues using RNeasy Plus Mini Kit (Qiagen). cDNA was prepared using the iScript cDNA Synthesis Kit (Bio-Rad). Quantitative PCR analysis was performed using the Power SYBR™ Green master mix (Applied Biosystems) in a thermocycler. Fold change in expression of target mRNA relative to β-actin (*ACTB)* mRNA was calculated using the delta-delta Ct method. Primer sequences used are as follows: *hBNIP3* – forward, 5′- AGCATGAGTCTGGACGGAGTAG −3′, reverse, 5′- CCTGTTGGTATCTTGTGGTGTCTG −3′; *hBNIP3L* – forward, 5′- GCACAACAACAACAACAACTG −3′, reverse, 5′- CATTGCCATTATCATTGCCATTG −3′; *hGLUT1* – forward, 5′- AGGACAAGTCCAGAACGAGAT −3′, reverse, 5′- AGCTACGCCTCGAAAATTAAACA −3′; *hACTB* – forward, 5′- CATGTACGTTGCTATCCAGGC-3′, reverse, 5′- CTCCTTAATGTCACGCACGAT-3′; *mBnip3 –* forward, 5′- GTGGTCAAGTCGGCCGG −3′, reverse, 5′- GCGCTTCGGGTGTTAAAGA −3′; *mBnip3l –* forward, 5′- CCTCGTCTTCCATCCACAAT −3′, reverse, 5′- GTCCCTGCTGGTATGCATCT −3′; *mGlut1 –* forward, 5′- TCTCGGCTTAGGGCATGGAT −3′, reverse, 5′- TCTATGACGCCGTGATAGCAG −3′; and *mActb –* forward, 5′- CTCTCCCTCACGCCATCCTG −3′, reverse, 5′- TCACGCACGATTTCCCTCTCAG −3′.

### Western blot

T84 cells were rinsed with PBS and collected and lysed in 200 µL of radioimmunoprecipitation assay (RIPA, 50 mM Tris-HCl pH 8.0, 1 mM EDTA, 1% Triton X-100, 10% SDS, 0.5% sodium deoxycholate, 150 mM NaCl) buffer with protease inhibitors on ice. Mouse colon samples were harvested into 350 µL of RIPA buffer and flash frozen in liquid nitrogen. Samples were homogenized by sonication, and insoluble materials removed by centrifugation at 10,000 g for 5 min at 4°C. Protein quantity of the supernatant was determined using the Bradford reagent. Laemmli sample buffer was added to each sample. Samples (20 µg) were run and separated on 8% SDS-PAGE gels and transferred to polyvinylidene fluoride (PVDF) membranes. Membranes were blocked overnight at 4°C in Tris-buffered saline with 1% Tween-20 (TBS-T) and 5% milk. Membranes were then incubated with primary antibodies in blocking buffer overnight at 4°C (rabbit anti-HIF-1α, 1:500, NB100-134, Novus Biologicals; rabbit anti-β-actin 1:10,000, ab8227, Abcam). Membranes were washed for four 10 min TBS-T washes before incubation with secondary antibodies in blocking buffer for 1 h at room temperature (peroxidase conjugated goat anti-rabbit IgG, 1:5000, MP Biomedicals). After four 10 min TBT-washes, the membranes were incubated with chemiluminescence detection solution and imaged on the ChemiDoc™ MP imager.

### Metabolite extraction and analyses

*Metabolites Extractions and HPLC-DAD*: Cell and colon tissue metabolites were extracted and analyzed as previously described with minor variations.^61,62^ The metabolites were detected by absorption at wavelengths of 210, 254, and 280 nm, with their absorbance spectra and retention times verified by co-injection with authentic standards. Metabolites for HPLC-ESI MS analyses were resuspended in deionized water (pH 7.2).

*HPLC-ESI MS*: Analyses were performed on an Agilent Technologies 1260 Infinity II LC/MSD iQ with electrospray ionization (ESI) mass detection. Negative ion mass-to-charge ratios (m/z) were scanned from 50 to 500. The metabolite extracts were chromatographed using a Sepax Carbomix column (Pb-Np5:8%, 5 μm, non-porous, 4.6 × 300 mm) (mobile phase A: water; mobile phase B: acetonitrile; column temperature, 75°C). Chromatographic separation was performed using a combination of isocratic and gradient methods, including column washing and equilibration periods at the end (0 min: 100% A, 0.12 mL/min; 60 min: 100% A, 0.12 mL/min; 70 min: 70% A, 0.5 mL/min; 145 min: 70% A, 0.5 mL/min; 150 min: 100% A, 0.5 mL/min; 164 min, 100% A, 0.5 mL/min; 165 min, 100% A, 0.12 mL/min; 170 min, 100% A, 0.12 mL/min). The metabolites were detected by the masses of their negatively charged ions (M-1 ± 0.03; 2-oxoglutarate, 145 m/z; succinate, 117 m/z; fumarate, 115 m/z; butyrate, 87 m/z), with their retention times and m/z verified by co-injection with authentic standards.

### Recombinant protein expression and purification

PHD2_181-402_ cDNA was cloned in a pET-28a (+) vector, and the plasmid was transformed into *E. coli* strain BL21 (DE3). Bacteria were allowed to grow at 37°C in M9 minimal media where^15^N-ammonium chloride was added to obtain^15^N-uniformly labeled protein. Protein expression was induced using 0.2 mM IPTG when an O.D. of 0.7 was reached, and then bacteria were grown at 18°C overnight. Bacteria pellets were harvested via centrifugation for 20 min at 4,000 x g. The lysate was purified using a HisTrap FF column (GE Healthcare), where the protein was eluted using high-imidazole buffer (25 mM HEPES, 200 mM NaCl, 250 mM imidazole, pH 7.5). The eluted protein (2–3 mg/mL) was incubated with 15,000-equivalents of EDTA at 4°C overnight. Further purification was done on a size-exclusion Superdex 75 HiLoad 16/600 column (GE Healthcare) to obtain PHD2_181-402_ in NMR buffer (Tris 50 mM, NaCl 20 mM, ZnCl_2_ 100 μM) at pH 6.5.^63^ The protein was dialyzed into the appropriate buffers for the following assays.

### PHD2_181-402_ inhibition assays

PHD2_181-402_ activity and inhibition assays were performed with the Succinate-Glo JmjC Demethylase/Hydroxylase Assay Kit (Promega) according to manufacturer protocol and as detailed in Alves *et al*. (2018).^24^ PHD2_181-402_ protein was dialyzed into 50 mM HEPES buffer pH 7.5. To delineate the optimal PHD2_181-402_ concentration to be used in the assays, serial two-fold dilutions of PHD2_181-402_ in 5 µL volumes were combined with 5 µL PHD2_181-402_ of enzyme reaction mix for a final reaction volume of 10 µL that contained PHD2_181-402_ starting from 2 µM to 2 nM and a 0 PHD2_181-402_ blank and PHD2_181-402_ enzyme reaction mixture containing 10 µM 2-OG, 10 µM HIF-1α_547-581_ peptide, 10 μM Fe (II), and 100 μM ascorbic acid in 50 mM HEPES pH 7.5 in 384-well white plates. The enzymatic reaction was incubated at room temperature for 10 minutes, after which succinate formation was detected according to manufacturer protocol. Briefly, 10 µL of Succinate Detection Reagent I was added to the samples and incubated for 60 min at room temperature, which stops the enzymatic reaction and converts produced succinate into ATP. Then, 20 µL of Succinate Detection Reagent II was added to the reactions and incubated for 10 min at room temperature before the luciferase-generated luminescence was recorded on a plate-reading luminometer. Succinate standard curves were used to determine the sensitivity and linear range of the bioluminescent succinate detection. Succinate standards were prepared in the PHD2_181-402_ enzyme reaction mixture containing 10 µM 2-OG, 10 µM HIF-1α_547-581_ peptide, 10 μM Fe (II), and 100 μM ascorbic acid in 50 mM HEPES pH 7.5. Succinate standards were serially diluted two-fold from 15 µM to 58.6 nM and a 0 succinate blank. 10 µL of succinate standards were used for detection as described above.

For determining the K_m_ of PHD2_181-402_ for 2-OG, 5 µL of PHD2_181-402_ were combined with 5 µL of serially two-fold titrated 2-OG in PHD2_181-402_ enzyme reaction buffer for a final reaction volume of 10 µL that contained 100 nM PHD2_181-402_ and PHD2_181-402_ enzyme reaction mixture containing 2-OG from 40 µM to 625 nM including a 0 2-OG blank, and 10 µM HIF-1α_547-581_ peptide, 10 μM Fe (II), and 100 μM ascorbic acid in 50 mM HEPES pH 7.5. Reactions were incubated at room temperature for 10 minutes and succinate detected as described above and rate of succinate formation calculated. K_m_ values were extracted from the data after fitting to the Michaelis–Menten least squares nonlinear regression in GraphPad Prism 9.

For obtaining IC50 values and assaying inhibition by different compounds (SCFAs, DMOG, etc.), 2.5 µL of serially two-fold titrated inhibitor compound was mixed with 2.5 µL of PHD2_181-402_ and incubated for 15 minutes. Then, 5 µL of PHD2_181-402_ enzyme reaction buffer was added to achieve a final reaction volume of 10 µL containing SCFAs and butyrate-derived compounds from 100 mM to 195.3 µM and blank and DMOG from 40 mM to 78.1 µM and blank with 1 µM PHD2_181-402_, 10 µM HIF-1α_547-581_ peptide, 10 μM Fe (II), and 100 μM ascorbic acid in 50 mM HEPES pH 7.5. Reactions were incubated at room temperature for 10 minutes and succinate detected. Percentage of PHD2_181-402_ activity were calculated by dividing amount of succinate produced by the amount of succinate produced in the blank inhibitor well, which was deemed as 100% activity. Measured IC50s were calculated from fitting the percentage activity data to the [Inhibitor] vs. normalized response – variable slope least squares nonlinear regression in GraphPad Prism 9. True IC50s were calculated from measured IC50s with the equation derived by Wu *et al*.^26^ describing the relationship between the measured IC50 of an inhibitor and the percentage of substrate conversion. For mode of inhibition experiments, butyrate was serially diluted two-fold from 100 mM to 195.3 µM and blank and DMOG from 40 mM to 78.1 µM and blank in the final 10 µL reaction volume with 1 µM PHD2_181-402_, 10 µM HIF-1α_547-581_ peptide, 10 μM Fe (II), and 100 μM ascorbic acid in 50 mM HEPES pH 7.5, and 2-OG was used at either 1, 5, 10, 40, or 100 µM in the final reaction. The intrinsic inhibitory constant (K_i_) of butyrate was calculated using the equation from Cer *et al*.^27^ with K_m_ for 2-OG, true IC50 value, PHD2_181-402_ and 2-OG concentration used in assays, and mode of inhibition.

Blank controls for PHD2_181-402_, HIF-1α_547-581_ peptide, Fe (II), and 2-OG were all conducted to show that all elements were necessary for enzymatic reactions to proceed.

### Nuclear Magnetic Resonance (NMR)

Following an established protocol, all NMR analyses were performed using PHD2_181-402_ and the diamagnetic element (Zn^2+^) rather than a paramagnetic element such as Fe^2+^ that would result in inhibitive signal reduction or loss due to fast relaxation induced by the unpaired electron.^63^

*Water-Ligand Observed via Gradient SpectroscopY (WaterLOGSY)*: Measurements were carried out on a 600 MHz triple-resonance Bruker cryoprobe spectrometer.^28^ Selective water inversion was achieved using an iBURP shaped pulse with 15 ms length and an inversion bandwidth of 0.4 ppm. The mixing time was set to 1 s, and the water was suppressed using double Watergate. 1024 scans were acquired with an interscan delay of 2 s. The sample concentration was 25 µM of PHD2_181-402_ mixed with 10 mM of the different ligands in NMR buffer (Tris 50 mM, NaCl 20 mM, ZnCl_2_ 100 μM) at pH 6.5.

*1D Adiabatic Fast Passage (AFP)-NOESY*: Experiments were conducted with the pulse sequency by Auer *et al*. (2010) on a 600 MHz triple-resonance Varian cryoprobe spectrometer.^29^ The experiment was run twice, with the position of the selective inversion pulse (iBURP) either applied at 0.75 or 2.02 ppm. The NOESY mixing time was adjusted to 400 ms, during which a Wurst 180 adiabatic pulse centered at water frequency was used to sweep over a range of 12 ppm. The strength of the Wurst 180 pulse was arrayed as −20, 15, 17, 20, 24, 28, 32, 36, and 38 dB to increase the contribution of the transverse cross relaxation to the overall cross-relaxation rate. The interscan delay was set to 2 s and 256 scans were acquired. The sample concentration was 25 µM of PHD2_181-402_ and 10 mM of butyrate in NMR buffer.

All NMR data were processed and visualized with NMRpipe and Sparky software.^64,65,66^

### Microscale Thermophoresis (MST)

Microscale thermophoresis (MST) was performed on a NanoTemper Monolith NT.115 Pico instrument (NanoTemper Technologies GmbH) at 25°C using auto-detect Pico Red at 20% excitation power. His-tagged PHD2_181-402_ was fluorescently labeled by incubating 100 μL of 200 nM protein solution in 50 mM HEPES pH 7.5 with 100 μL Red-tris-NTA 2^nd^ generation dye (100 nM) for 30 min. The reaction mixture was centrifuged for 10 min at 4 °C and 15,000 g speed. 25 nM of the protein and 16 two-fold dilution series of butyrate were loaded into sixteen standard capillaries (NanoTemper Technologies GmbH; the highest concentration was 75 mM). In the competition assay with 2-OG, a fixed concentration of 500 nM was added. The sigmoidal curves obtained were analyzed to extract the K_D_ value using NanoTemper Technologies GmbH analysis software.

### Statistical analysis

Data are expressed as mean values ± SEM. Statistical significance between two groups was evaluated with unpaired, two-way Student's t-test and between multiple groups was evaluated with 1-way ANOVA with Fisher’s Least Significant Different (LSD) test for multiple comparisons. Nonlinear regressions were performed as noted. Axll replicates were biological replicates and measurements were taken from distinct samples. A *p* value of less than 0.05 was considered significant (GraphPad Prism 9).

## Supplementary Material

Supplemental Material

## Data Availability

All data generated in this study are included in this article and supplementary information or are available from the corresponding author on reasonable request.
