# Supplementary material for: Microbiota-derived butyrate is an endogenous HIF prolyl hydroxylase inhibitor
Source: Gut Microbes. 2021 Jun 30;13(1):1938380. doi: 10.1080/19490976.2021.1938380 (PMC8253137; doi:10.1080/19490976.2021.1938380)
Supplement: Supplemental Material [file KGMI_A_1938380_SM1188.docx]

**Supplemental Information**

**
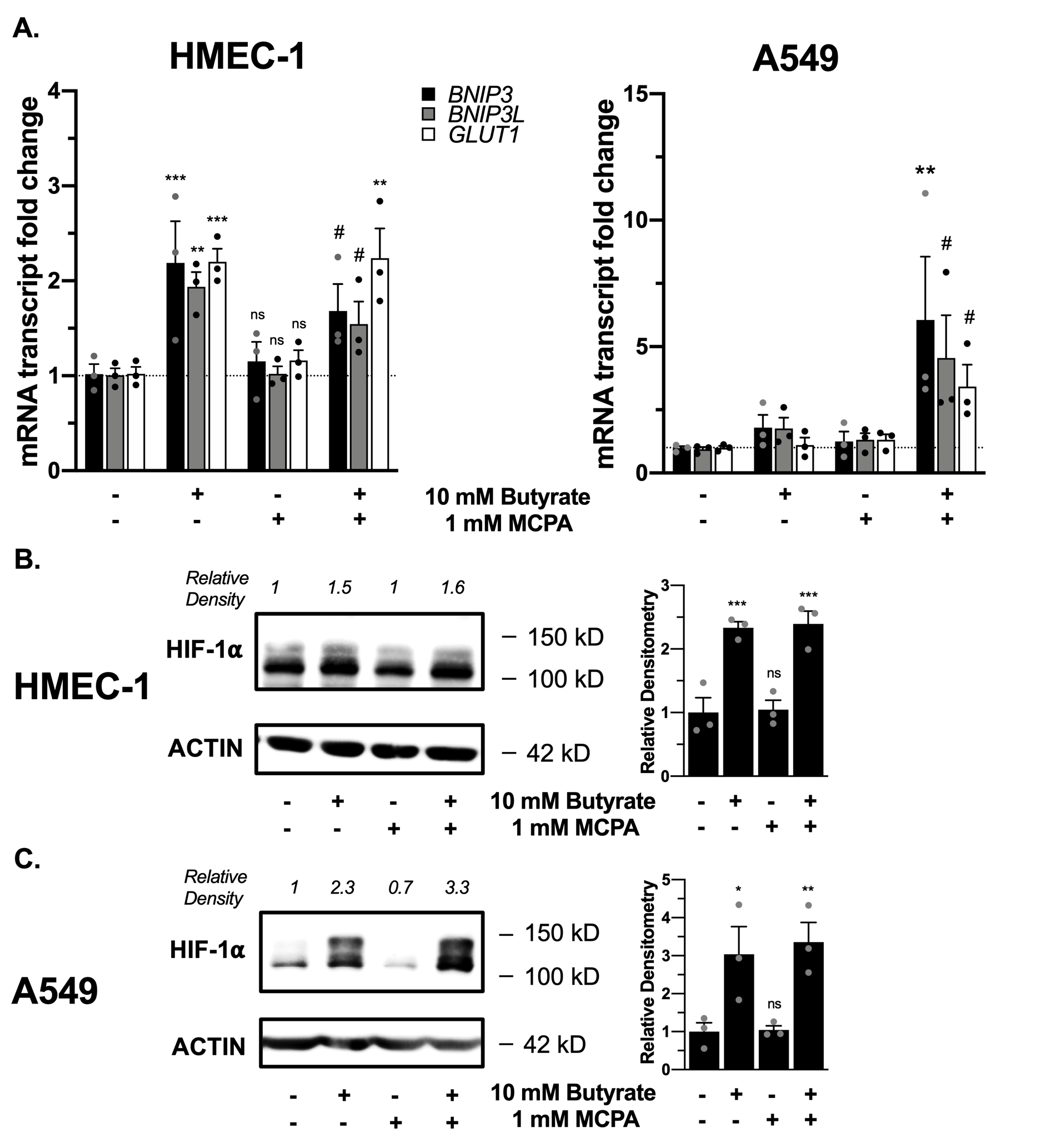
**

**Supplemental Figure 1.** *Butyrate stabilizes HIF independent of oxygen consumption in HMEC-1 and A549 cells.* (A) HIF-1α target mRNA expression in HMEC-1 and A549 cells treated with 10 mM butyrate with or without 1 mM MCPA, 1 mM DMOG, or 30 μM IOX2 for 4 h (*n*=3, error bars: SEM, *ns* not significant, # *p*<0.1, * *p*<0.05, ** *p*<0.01, *** *p*<0.001by 1-way ANOVA, Fisher’s multiple comparison). (B) HIF-1α protein expression in HMEC-1 and corresponding quantified densitometry (*n*=3, error bars: SEM, *ns* not significant, *** *p*<0.001by 1-way ANOVA, Fisher’s multiple comparison). (C) A549 cells treated with 10 mM butyrate with or without 1mM MCPA for 4 h. (*n*=3, error bars: SEM, *ns* not significant, * *p*<0.05, ***p*<0.01, by 1-way ANOVA, Fisher’s multiple comparison).

**
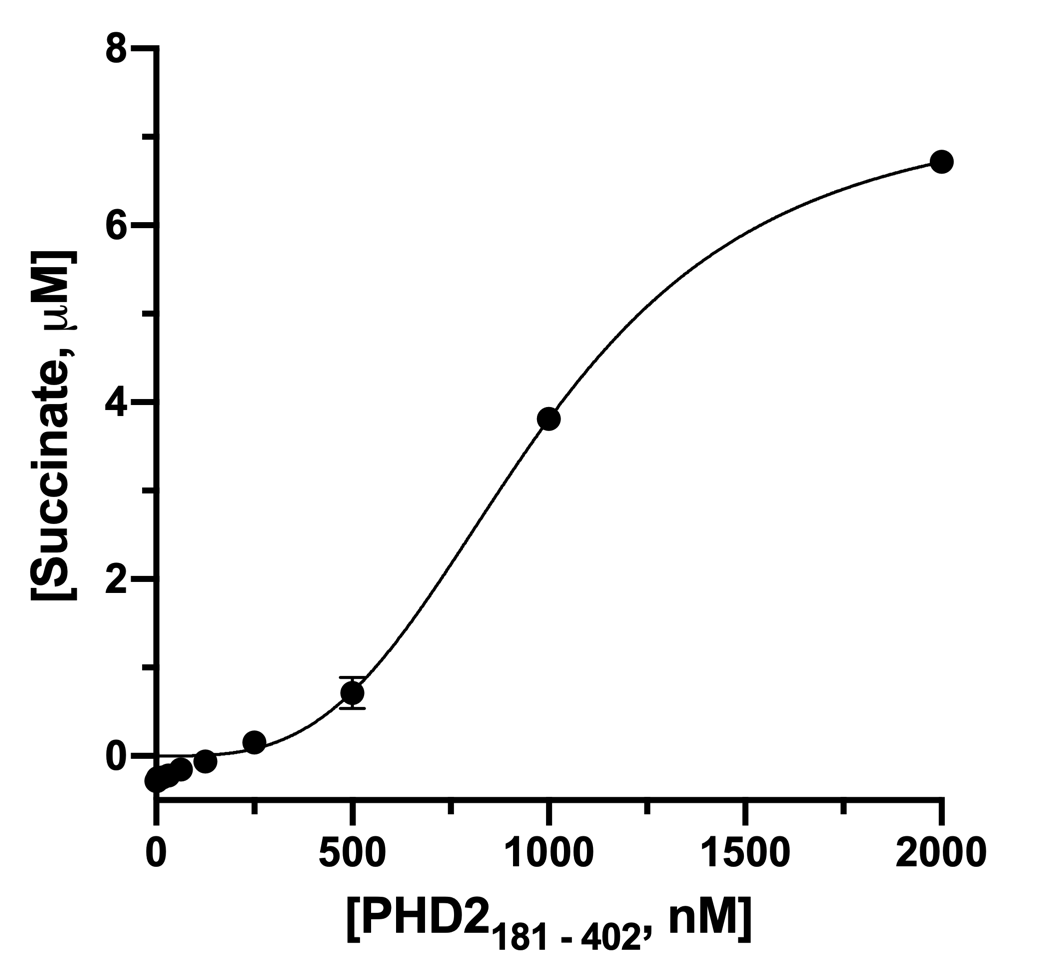
**

**Supplemental Figure 2.** *PHD2_181-402_ concentration curve for bioluminescent succinate detection assay.* Succinate levels were measured after incubating varying concentrations of PHD2_181-402_ from 2 nM to 2 μM with 10 μM 2-OG, 10 μM HIF-1α_547-581_ peptide, 10 μM Fe (II), and 100 μM ascorbic acid for a 10-minute reaction (*n*=3, error bars: SEM, Allosteric sigmoidal least squares fit).
